# Tropical cyclone simulations over Bangladesh at convection permitting 4.4 km & 1.5 km resolution

**DOI:** 10.1038/s41597-021-00847-5

**Published:** 2021-02-16

**Authors:** Hamish Steptoe, Nicholas Henry Savage, Saeed Sadri, Kate Salmon, Zubair Maalick, Stuart Webster

**Affiliations:** Met Office, FitzRoy Road, Exeter, EX1 3PB UK

**Keywords:** Natural hazards, Atmospheric dynamics

## Abstract

High resolution simulations at 4.4 km and 1.5 km resolution have been performed for 12 historical tropical cyclones impacting Bangladesh. We use the European Centre for Medium-Range Weather Forecasting 5^th^ generation Re-Analysis (ERA5) to provide a 9-member ensemble of initial and boundary conditions for the regional configuration of the Met Office Unified Model. The simulations are compared to the original ERA5 data and the International Best Track Archive for Climate Stewardship (IBTrACS) tropical cyclone database for wind speed, gust speed and mean sea-level pressure. The 4.4 km simulations show a typical increase in peak gust speed of 41 to 118 knots relative to ERA5, and a deepening of minimum mean sea-level pressure of up to −27 hPa, relative to ERA5 and IBTrACS data. The downscaled simulations compare more favourably with IBTrACS data than the ERA5 data suggesting tropical cyclone hazards in the ERA5 deterministic output may be underestimated. The dataset is freely available from 10.5281/zenodo.3600201.

## Abstract (summary)

**Table:** 

Measurement(s)	atmospheric wind speed • wind speed of gust • temperature of air • humidity • geopotential height • water-based rainfall • snowfall • pressure of air • air pressure at sea level • wet bulb potential temperature • net down surface SW flux • surface downwelling SW flux in air
Technology Type(s)	computational modeling technique
Factor Type(s)	spatial resolution: 4.4 km & 1.5 km
Sample Characteristic - Environment	atmospheric weather • atmospheric boundary layer • cyclone
Sample Characteristic - Location	Bangladesh • South Asia

Machine-accessible metadata file describing the reported data: 10.6084/m9.figshare.13560560

## Background & Summary

To construct this dynamically simulated tropical cyclone dataset we use the latest generation Met Office regional model to simulate tropical cyclones (TCs) over the Bay of Bengal (BoB) at grid-box resolutions of 4.4 km and 1.5 km. Using the ERA5 reanalysis data^1,2^ to initialise and provide boundary conditions for our regional models, we dynamically downscale 12 historical TCs that made land-fall over Bangladesh between 1991 and 2019, using an ensemble approach.

Downscaling of ERA5 is reported in a few other studies: Bonanno *et al*.^3^ downscale ERA5 using the Weather Research and Forecasting (WRF) model to produce a new 7 km reanalysis over Italy; preliminary work by Taddei *et al*.^4^ use ERA5 to force the BOlogna Limited Area Model-MOdello LOCale (BOLAM-MOLOCH) regional model for the purposes of coastal risk assessment in the North Western Mediterranean sea, and Wang *et al*.^5^ use ERA5 to run a 10 km WRF domain over high mountain Asia. Specifically examining tropical cyclones, many studies use variations of the Weather Research and Forecasting (WRF) Model^6^, such as Kaur *et al*.^7^ who use WRF to downscale the National Center for Environment Prediction (NCEP) Climate Forecast System (CFSv2) and its atmospheric component Global Forecast System (GFS) to 9 km over the north Indian Ocean for two historical cases (Mora and Ockhi), with analysis focusing on the spatial accuracy of rainfall and 850 hPa vorticity, and the vertical profiles of wind and temperature. They conclude that the downscaled model significantly improves the spatial distribution of rainfall, maximum vorticity evolution, wind, and temperature profiles for mature phase cyclones. Studies specifically examining the BoB simulations^8–10^ typically make empirical comparisons of TC simulations at ~10 km resolution against observationally based data, but often with an India-centric domain that contains a larger number of landfalling events. By contrast, in this study we specifically focus on Bangladesh, with simulations at higher resolution.

We make 12 variables available, including: air temperature, maximum wind gust speed, minimum air pressure at sea level and precipitation amounts (see Table 1), at a range of temporal scales (including model instantaneous values) as well as hourly and daily aggregations. Simulations are performed for the following tropical cyclones (landfall date): BOB01 (Apr 1991), BOB07 (Nov 1995), TC01B (May 1997), Akash (May 2007), Sidr (Nov 2007), Rashmi (Oct 2008), Aila (May 2009), Viyaru (May 2013), Roanu (May 2016), Mora (May 2017), Fani (May 2019) and Bulbul (Nov 2019). Table 2 lists approximate landfall times and their International Best Track Archive for Climate Stewardship (IBTrACS)^11,12^ ID number. Note that for this paper, the name refers to a shorthand identifier, used for file naming purposes, but it does not necessarily reflect the official storm identifier. At the time of writing, ERA5 data was only available from 1979 onwards, so our new catalogue excludes cyclones prior to 1979, most notably Cyclone Bhola of November 1970.

**Table 1:** Available model output, their file name identifier, SI units and, where applicable, the levels at which data is available.

Variable	Identifier	Unit	Pressure Levels (hPa)	Height from surface (m)
net down surface SW flux corrected	rsnds	W m^−2^		
wet bulb potential temperature	wbpt	K	200, 300, 500, 700, 850	
air pressure at sea level	psl	Pa		
air temperature	tas	K		1.5
geopotential height	zg	M	200, 300, 500, 700, 850	
relative humidity	hur	%	200, 300, 500, 700, 850	1.5
rainfall amount	prlst	kg m^−2^		
snowfall amount	prlssn	kg m^−2^		
surface downwelling SW flux in air	rsds	W m^−2^		
wind speed of gust	fg	m s^−1^		10
x wind	ua	m s^−1^	200, 300, 500, 700, 850	10
y wind	va	m s^−1^	200, 300, 500, 700, 850	10

**Table 2:** List of tropical cyclones downscaled in this dataset. IBTrACS ID refers to the International Best Track Archive for Climate Stewardship storm identifier.

Name	Landfall Date (DD/MM/YYYY HH:MMZ)	IBTrACS ID
BOB01	30/04/1991 00:00Z	1991113N10091
BOB07	25/11/1995 09:00Z	1995323N05097
TC01B	19/05/1997 15:00Z	1997133N03092
Akash	14/05/2007 18:00Z	2007133N15091
Sidr	15/11/2007 18:00Z	2007314N10093
Rashmi	26/10/2008 21:00Z	2008298N16085
Aila	25/05/2009 06:00Z	2009143N17089
Viyaru	16/05/2013 09:00Z	2013130N04093
Roanu	21/05/2016 12:00Z	2016138N10081
Mora	30/05/2017 03:00Z	2017147N14087
Fani	04/05/2019 06:00Z	2019117N05088
Bulbul	09/11/2019 18:00Z	2019312N16088

Landfall dates are provided for reference and do not necessarily reflect the landfall date of the downscaled data. Similarly, names are provided as a shorthand identifier, and are used for file naming purposes, but do not necessarily reflect the official storm identifier.

Whilst this data descriptor paper focuses on validating this new model dataset, a companion paper^13^ uses the data presented here to produce spatially-consistent exceedance probability estimates for extreme gust speeds over Bangladesh, and demonstrates a method for producing a transparent decision-making framework for tropical cyclone warnings

## Methods

### Numerical modelling

**Fig. 1:**
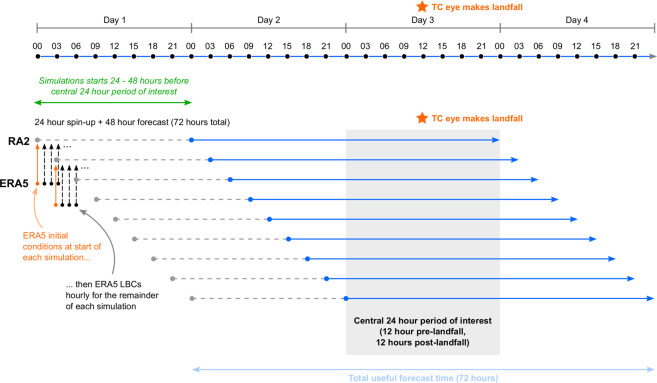
Ensemble configuration for the RAL2-T (UM) downscaling suite. ERA5 initial conditions (orange dots) initialise the simulation start point (grey dots). Each ensemble member then has a 24 hour spin-up period (grey dashed lines) which is discarded from all analysis. The 48-hour simulation that is kept is represented by the solid blue line. ERA5 lateral boundary conditions (LBCs, black dots) feed into the 4.4 km domain every hour. The lagged ensemble is designed to simulate a central 24-hour period (shaded grey), common to all ensemble members and centred on the tropical cyclone land-fall time (orange star), but also sample a range of ERA5 initial conditions.

Our high-resolution convection-permitting modelling utilises the latest generation Met Office Unified Model^14^ v11.1, regional atmosphere configuration RAL2-T, a further development of RAL1-T^15^ – hereafter referred to as RAL2. For each historical tropical cyclone case listed in Table 2, we run the RAL2 model in a ‘downscaling’ configuration, using ERA5 data to initialise and provide boundary conditions for a series of 9 time-lagged ensembles (see Fig. 1 for a visual representation of this configuration).

As there is no data assimilation process or nudging, the initial conditions imposed by ERA5 are found to have significant influence on the resulting tropical cyclone development. The time-lagged configuration is designed to limit the free-running model time to 72 hours, whilst ensuring that the central 24-hour period of interest (centred on the tropical cyclone landfall time) is sufficiently sampled from a range of ERA5 initial conditions. This initial condition ensemble approach produces a set of 9 plausible tropical cyclone development scenarios associated with each named event. After initialisation, each ensemble member is free running for 72 hours, with hourly boundary conditions provided by ERA5. Each run requires a 24-hour spin-up period as the regional model adjusts from the weak initial state inherited from the ERA5 driving global model. This initial 24 hours of model data are discarded in subsequent analysis and data files. Together, the amassed ensemble provides 9 simulations of the central 24 hours, but covers a total period of 72 hours.

**Fig. 2:**
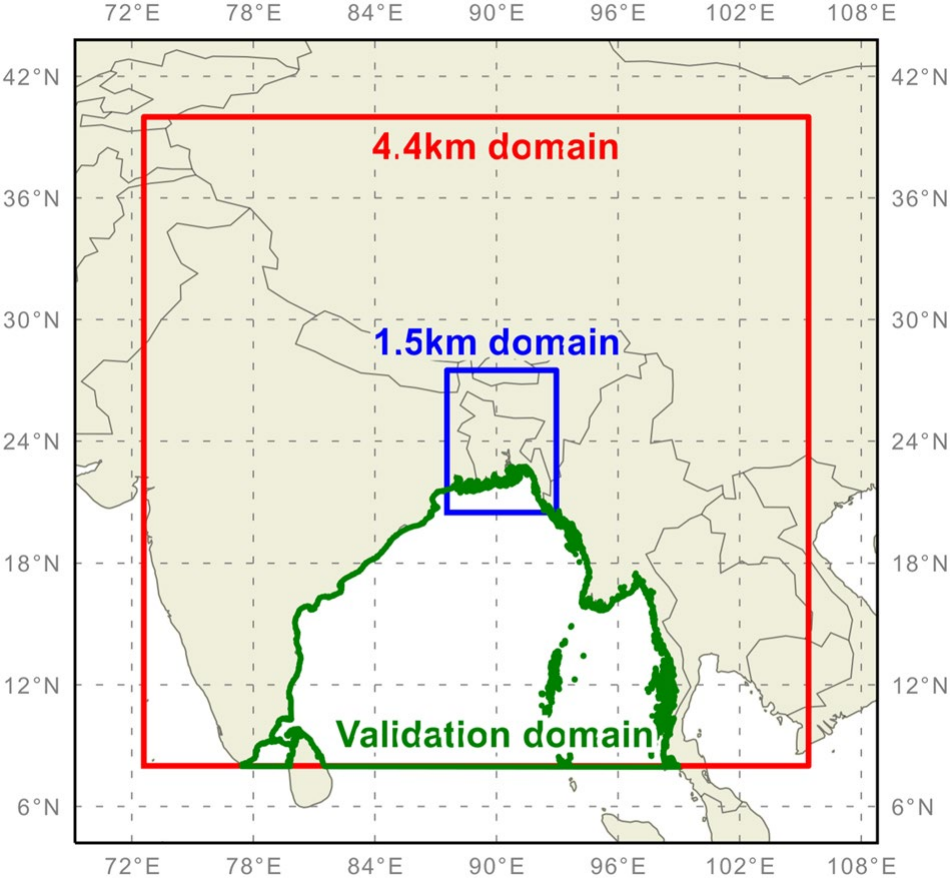
Model domains used for the 4.4 km (red) and 1.5 km (blue) regional models. ERA5 data, with global coverage, provides initial conditions for the 4.4 km domain. The 1.5 km model takes its initial and boundary conditions from the 4.4 km model. The domain data mask used for validation plots on Section 3 is in green.

The RAL2 4.4 km domain avoids placing model boundaries over the Himalayas and covers Nepal, Bhutan, Myanmar, most of India, and parts of the Tibetan plateau; the RAL2 1.5 km domain is limited to Bangladesh only (Fig. 2). To ensure model stability over this mountainous terrain, the RAL2 model was run with a 30 second time-step for both 4.4 km and 1.5 km simulations with additional orographic smoothing applied (using a 1-2-1 filter) to model cells 1500 m above mean sea level.

### Storm tracking

Storm tracking is performed on 3-hourly fields of RAL2 mean sea-level pressure (MSLP), 400 hPa temperature and 10 m wind speed, using the Tempest extremes software^16^. Unfortunately, 3-hourly fields are not frequent enough to estimate landfall time using the Tempest tracking algorithm for RAL2 data. The tracking algorithm has two parts – the initial feature detection and the stitching of these features to calculate tracks.

Feature detection is based on finding minima in air pressure at sea level, with features within a radius of 6° of each other being merged. The features are then further refined with a two ‘closed contour criteria’. First an increase in sea level pressure of at least 200 Pa (2 hPa) within 5.5° of the candidate node, and second a decrease in 400 hPa air temperature of 0.4 K within 8° of the node within 1.1° of the candidate with maximum air temperature.

Stitching, to combine the individual features into tracks, uses a maximum distance between features of 3°, a minimum track length of 2 points (equivalent to 6 hours) and a minimum path distance of 0.1°. We also apply a topographic filter and a filter on maximum wind speed: tracks were rejected if they did not have at least one time-step and last at least 24 hours at an altitude less than 10 m; and if they did not have maximum wind speed of at least 17 m s^−1^ at one time-step.

### Datasets

ERA5^1,2^ is the fifth and latest generation reanalysis dataset issued by the European Centre for Medium-Range Weather Forecasts (ECMWF). It combines both model data and observations on a real-time basis in a data assimilation process. Like a forecast, newly available observations are combined with model data to produce the best estimate of the state of the atmosphere. ERA5 data offers many improvements on the previous reanalysis, ERA-Interim, including more developed model physics and dynamics and an increased horizontal resolution of 30 km. In term of vertical resolution and extent, it has 137 model levels up to 80 km. For ERA5, we compare our simulated storm data with ‘10 metre wind gust since previous post-processing’ defined as the maximum 3-second wind for each hour (parameter ID 49) and MSLP (parameter ID 151). Prior to 30th Sep 2008, ERA5 gust estimates only include turbulent contributions; the convective contribution was added to the wind gusts in post-processing for events after this date^17^.

International Best Track Archive for Climate Stewardship^11,12^ forecasts are made by numerous forecasting centres around the world, and consists of the positions and intensities of tropical cyclones^18^. For our validation purposes, two Regional Specialized Meteorological Center (RSMC) datasets are used: the India Meteorological Department, New Delhi (IMD), and the Central Pacific Hurricane Center, Honolulu (CPHC). IBTrACS best track data are typically calculated using a post-season reanalysis of storm positions and intensities from all available data, including ship, surface and satellite observations^18^. Typically, best track data consist of a time series of the storm’s position, maximum sustained wind speed (in knots) and minimum central pressure. Estimated uncertainty of the IBTrACS forecast wind speed are ± 10 to ± 20 knots, with positional uncertainty radiuses of 10 km to 40 km, dependent on wind speed intensity^19^. No uncertainty information is provided for pressure, but we note that the World Meteorological Organisation typically assume reporting precision of ± 3 hPa. We also note that IBTrACS data is subject to forecaster best judgement and best track data typically lags the provisional operational data cyclone estimates by some months, subject to the availability of reanalysis data.

For the IBTrACS dataset we compare with ‘maximum sustained wind speed’ and MSLP. Although the World Meteorological Organisation^20^ defines sustained wind speed as a 10-minute average windspeed at 10 m height above ground, it is reported as 1-minute averages by US forecast centres, and 3-minute averages by IMD. Some agencies, including CPHC, estimate gust speeds; however this data is not available for the BoB basin. Methods for obtaining maximum wind speed in IBTrACS vary by agency, as do their availability of TC observation data. IBTrACS minimum central pressure is generally estimated with both subjective and objective satellite analysis as well as automated buoys that may be present^19^. Note that IBTrACS estimates usually end once the cyclone makes landfall.

### Comparing datasets

For the purposes of comparing RAL2 simulated winds and gusts with IBTrACS and ERA5, the RAL2 maximum sustained wind speed is taken as the maximum of a single RAL2 model timestep windspeed over the accumulation period (1 hour). This is broadly comparable to a sustained maximum windspeed calculated with 30-second averaging period. In contrast, the parameterised RAL2 gust diagnostic represents a prediction of the 3-second average windspeed at every timestep. The maximum of this 3-second average speed over an hour is then taken to give the hourly maximum 3-second gust speed.

Considering the ERA5 and RAL2 model physics, ERA5 uses a mass flux scheme for cumulus parameterisation^21^ whereas RAL2, while not truly resolving deep convection, is able to explicitly represent deep convective processes within the resolved dynamics. At these kilometre-scale resolutions the lower horizontal size limit of convective cells is still set by the effective resolution (5 to 10 times the grid length^22,23^). More generally, only grid spacings on the order of 1 km are comparable to the size of particularly energetic eddies in the planetary boundary layer^24^, so the turbulent processes as well as the dominant turbulent length scale will be under resolved in both our downscaled model and ERA5. ERA5 gusts are parametrised based on the 10 m wind speed, friction velocity, atmospheric stability, roughness length and a convective contribution based on wind shear between the model levels at 850 hPa and 925 hPa^17^. It is known that extreme gusts associated with vigorous convection in ERA5 are generally under-estimated, sometimes by a factor of two^25^. The RAL2 model uses a gust parametrisation based on 10 m wind speed with scaling proportional to the standard deviation of the horizontal wind that also accounts for friction velocity, atmospheric stability and roughness length^26^.

Comparisons of minimum MSLP are more straightforward. We compare the RAL2 hourly minimum MSLP estimated every 30-seconds, with the hourly minimum MSLP from ERA5, and the 3-hourly minimum MSLP from IBTrACS.

## Data Records

Our RAL2 model output is available from Zenodo in NetCDF format^27^. All data is licenced under Creative Common Attribution 4.0 International (CC BY 4.0).

The ERA5 data used to drive our RAL2 model and used for validation is available from the Copernicus Climate Change Service portal https://climate.copernicus.eu/climate-reanalysis.

The IBTrACS version 4 data used for validation is available from https://www.ncdc.noaa.gov/ibtracs/index.php?name=ib-v4-access.

To facilitate integration with loss modelling processing necessary for risk management and risk transfer, we also make our RAL2 data available in a format compatible with the open source Oasis loss model^28^. This data format is designed to be used as one component of a loss model and is formed of CSV and binary files. This data is available under CC-BY 4.0 licence from https://oasishub.co/dataset/bangladesh-tropical-cyclone-historical-catalogue.

## Technical Validation

A lack of reliable, high-frequency and consistent meteorological observation data available for Bangladesh mean that verification of modelling results against *in-situ* observational data is not possible. Instead we establish the validity of the RAL2 4.4 km data relative to ERA5 and the IBTrACS catalogue. It is important to recognise the differences in how the data are collected, their processing and resolution (see Table 3). Comparison of storm tracks is performed against the IBTrACS best track data only.

**Table 3:** Datasets and their key characteristics used in the model validation.

Dataset	Data Type	Spatial Resolution	Temporal Resolution	Compared Variables	Convective/parameterised wind speed
Downscaled (RAL2) model data	Gridded	4.4 km	1-hourly	Gust, Wind, MSLP	Convective permitting
Downscaled (RAL2) model data	Gridded	1.5 km	1-hourly	Gust, Wind, MSLP	Convective permitting
ERA5	Gridded	30 km	1-hourly	Gust, Wind, MSLP	Parameterised
IBTrACS v4, US	Time Series	10 km (0.1°)	3-hourly	Wind, MSLP	Observed from various sources
IBTrACS v4, India	Time Series	10 km (0.1°)	Interpolated to 3-hourly (most data reported at 6 hourly)	Wind, MSLP	Observed from various sources

For the purposes of validation, we focus on three key variables: maximum wind speed, maximum gust speed and minimum pressure at mean sea level (MSLP). We have a particular interest in gust speed, as these are strongly related to storm damage^29,30^ and are commonly used within the catastrophe modelling industry. All comparisons against IBTrACS compare hourly maximum wind from our RAL2 4.4 km model versus 3-hourly maximum wind speed estimates from IBTrACS. For maximum gust speed, we compare the RAL2 hourly maximum 3-second gust diagnostic with ERA5 hourly maximum 3-second gust speed diagnostic. MSLP estimates are comparable across all three datasets. In each case, the comparison is performed over a land-masked longitude-latitude domain that extends [79, 100]°E and [10, 25]°N – see Fig. 2. This domain explicitly seeks to focus on the Bay of Bengal so as to compare model fields without land effects. In all cases, excluding land areas has very minor impact on the validation comparison (not shown) as peak wind, gust and minimum MSLP all occur over the ocean. Although our storm tracking output does not allow us to explicitly compare the time of landfall between datasets, we expect that differences in the time of peak wind speeds would be mirrored in the differences in the time of landfall across datasets as RAL2 peak wind speeds tend to occur just prior to landfall.

**Fig. 3:**
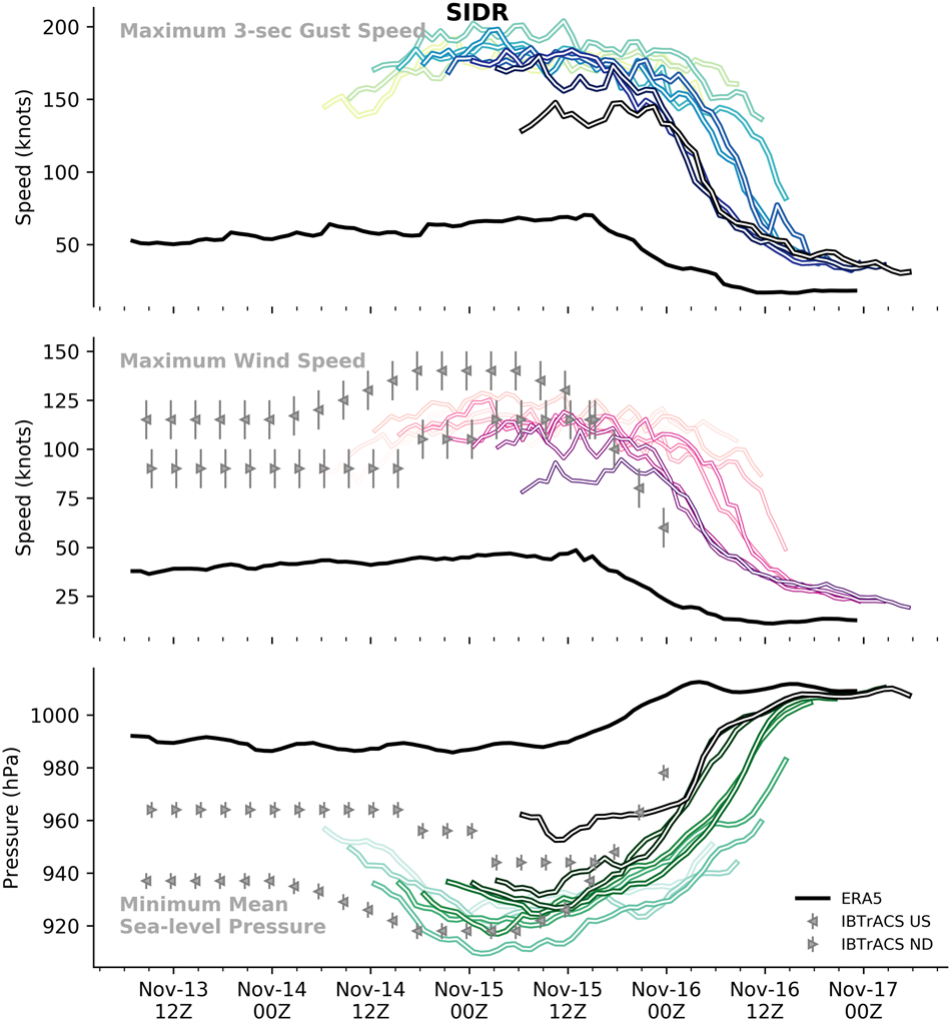
Storm specific comparison of maximum gust speed (top), maximum wind speed (middle) and minimum sea-level pressure (bottom) for tropical cyclone Sidr (Nov 2007). The dynamically downscaled, high-resolution Met Office model (RAL2) is shown by the coloured lines, where each individual line represents one ensemble member, where the initialisation time is coloured lighter to darker. These are shown against IBTrACS (grey triangles with uncertainty ranges) and ERA5 (black line). Equivalent plots for other events can be found in Appendix B.

Each validation plot (Fig. 3, and Supplementary Information Figures A1–A11) displays the gust speed, wind speed and MSLP from the ERA5, IBTrACS and RAL2 4.4 km. We resample the IBTrACS 3-hourly data by forward filling to 1-hourly intervals to aid the comparison of max/min timing with ERA5 and RAL2 datasets. Where IBTrACS maxima (minima) persist over several hours, the time differences reported in Sections 4.1 and 4.2 are then the minimum time difference between padded IBTrACS data and RAL2. The actual difference of RAL2 with respect to ERA5 (RAL2 – ERA5) is denoted ΔERA5 for brevity. For IBTrACS, actual differences with respect to IMD and CPHC are denoted ΔND and ΔUS respectively.

The statistical robustness of differences between datasets are assessed using the percentile bootstrap hierarchical shift function^31–34^. Given the potential skewness of the data, rather than looking at the differences of a single estimate of central tendency across all events (e.g. the median), differences are assessed for deciles (or percentiles) across the full distribution of the data, calculated using the distribution-free Harrell-Davis estimator^35^. This method explicitly deals with the hierarchical setting of data representing the same event, sharing common synoptic atmospheric conditions, but where different events are independent in time. The robustness of differences is assessed using bootstrapped (n = 1000) uncertainty intervals for each decile difference. Where the 95% highest density interval (HDI) of uncertainty does not intersect zero, decile differences are considered statistically robust.

### Intensity and timing of maximum sustained wind speed

**Fig. 4:**
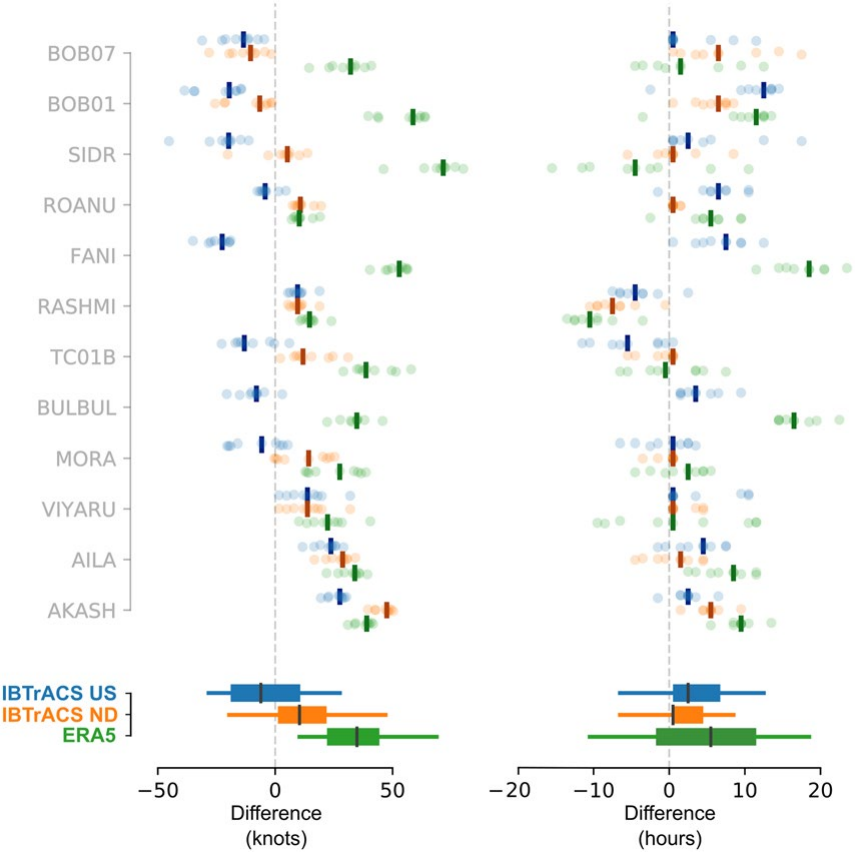
Differences in maximum wind speed intensity (left) and timing of maximum (right) for IBTrACS US (blue) ND (orange) and ERA5 (green) relative to RAL2 ensemble members, ordered by magnitude of the intensity difference. Comparisons are made only within the period of RAL2 data, up to 36 hours pre and post landfall. Differences are calculated relative to RAL2 maximum, such that a positive intensity (time) difference indicates that the RAL2 model is faster (ahead) of the respective ERA5 or IBTrACS data. IBTrACS data is resampled by forward padding data to hourly intervals to aid comparison with RAL2. Where there are joint maxima in the IBTrACS data over multiple timesteps, we plot the smallest differences. Individual model differences are shown by coloured circles, with median difference per storm are show by coloured bars. Lower boxplots aggregate differences across all storms, with the 50^th^ percentile marked by the black bar and whiskers extending to the 5^th^ and 9^th^ percentiles of the data.

For all events, RAL2 maximum sustained wind speeds are faster than ERA5 wind speeds (Fig. 4), with median (across all events) ΔERA5 = 35 kn (18 m s^−1^), with the 5^th^ to 95^th^ percentiles of the data spanning [10, 70] kn ([5, 36] m s^−1^). Comparing IBTrACS, median ΔUS = −6 kn (−3 m s^−1^) and ΔND = 10 kn, (5 m s^−1^). Assessing the robustness of differences, the distribution of ΔERA5 is robustly slower than RAL2 across all deciles (based on 95% HDI for each decile difference). ΔND is also robustly slower for differences greater than the 40^th^ percentile; however, note that at the time of writing, IBTrACS IMD maximum sustained wind speed data for Fani and Bulbul were unavailable. Although IBTrACS US data has a tendency toward faster sustained wind than RAL2 (i.e. negative ΔUS) these differences are not robustly different to zero at the 95% HDI.

The timing of maximum wind speed shows significant variation between events, with no clear correlation to peak wind intensity differences; however, generally RAL2 peaks are delayed relative to ERA5 and IBTrACS data. Across all events, median ΔERA5 = 5.5 hours delay, with ΔUS = 2.5 hours and ΔND = 0.5 hours. Only ΔERA5 and ΔUS times are robustly different to RAL2 (evaluated at the 95% HDI). The largest time differences occur against ERA5 data: e.g. for Fani, some RAL2 ensemble members show maximum wind intensities delayed by over 20 hours relative to ERA5, but it is noted that for these cases the ERA5 tropical cyclone simulation seems especially weak (for maximum wind, gust and minimum MSLP) compared to IBTrACS data. Some of the variance in peak times will also derive from the differences in data frequency (1-hourly for RAL2 versus 3-hourly for IBTrACS) but this requires further investigation to quantify.

### Intensity and timing of mean sea-level pressure

**Fig. 5:**
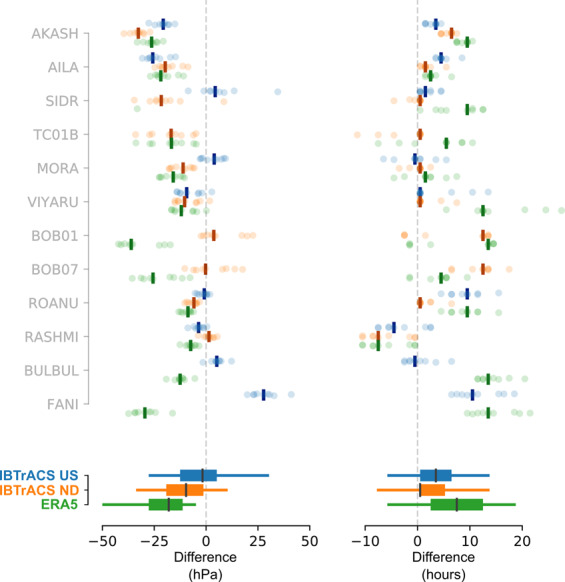
Differences in minimum MSLP (left) and time of minimum (right) for IBTrACS US (blue) ND (orange) and ERA5 (green) relative to RAL2 ensemble members, ordered by magnitude of the MSLP intensity difference. Details as for Fig. 4. A negative (positive) difference in MSLP indicates that the RAL2 MSLP minima are deeper (shallower) than the respective ERA5 or IBTrACS data. Note that IBTrACS ND MSLP data was not available for Fani or BulBul, and US MSLP not available for BOB01, BOB07 and TC01B, at the time of writing.

For most events, the RAL2 ensemble produces deeper MSLP minima than the ERA5 and IBTrACS data (Fig. 5), but whilst ΔERA5 (median = −18 hPa) and ΔND (median = −10 hPa) differences with RAL2 are robustly different to zero, ΔUS (median = −2 hPa) is not (all evaluated at the 95% HDI). At the time of writing, IBTrACS MSLP data for Fani and Bulbul are unavailable from IMD, and BOB01, BOB07 and TC01B are unavailable from CPHC.

As for wind speeds, the timing of RAL2 MSLP minimum is typically delayed relative to IBTrACS or ERA5 data. Median time difference of MSLP minima are similar to wind speed maxima differences: ΔERA5 = 7.5 hours delay, ΔUS = 3.5 hours and ΔND = 0.5 hours. Again, only ΔERA5 and ΔUS times are robustly different to RAL2 (evaluated at the 95% HDI). As for the timing of gust peaks (Section 3.1), the RAL2 simulation of Fani shows median delays in MSLP minima of 14 hours (ΔERA5) and 11 hours (ΔUS). BOB01 also has an equivalent delay of 13 to 14 hours (ΔND and ΔERA5 respectively).

### Intensity and timing of maximum 3-second gust speed

**Fig. 6:**
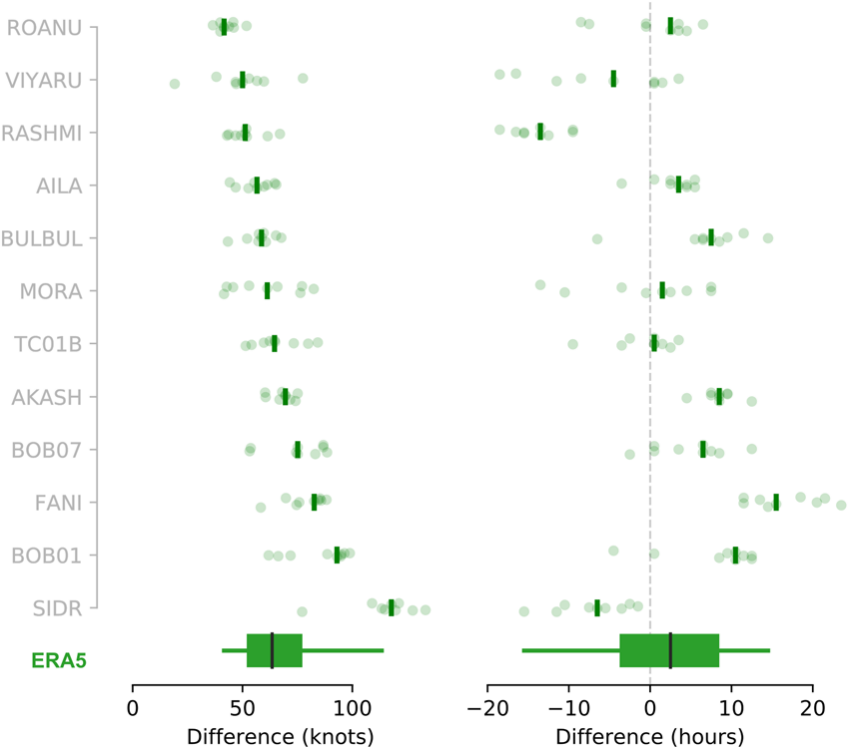
Differences in maximum 3-second gust speed (left) and timing of maximum gust speed (right) for ERA5 relative to RAL2 ensemble members, ordered by the magnitude of the gust speed difference. Details as for Fig. 4. A positive (negative) difference in gust speed indicates that the RAL2 gust speed maximum is faster (slower) than ERA5 data. Note that at the time of writing gust speed data was not available from IBTrACS for any of these events.

The distribution of RAL2 gust speeds across events, are uniformly higher than ERA5 (Fig. 6). The median difference across all events is 63 kn (32 m s^−1^, Fig. 6), with some particularly strong individual events showing median differences up to 93 kn (48 m s^−1^, BOB01) and 118 kn (61 m s^−1^, Sidr). Comparing differences in the RAL2 and ERA5 gust speed distributions using bootstrapped median difference by percentile across all events, shows that these differences are robustly different to zero at the 95% HDI.

As with wind and MSLP, differences in the timing of maximum 3-second gust speed vary considerably between events with no clear correlation between the magnitude of the gust difference and the absolute time differences. The median time difference across all events is 2.5 hours (Fig. 6), but this is not robustly different to zero at the 95% HDI.

### Storm tracks

We compare the track density of our nine downscaled ensemble members to IBTrACS in 30 × 30km spatial bins. Typically, the area influenced by the tropical cyclone wind hazard is in excess of 200 × 200 km, so this assessment of storm tracks plays a more important role in evaluating storm surge, primarily influenced by the area of low pressure at the centre of the cyclone.

**Fig. 7:**
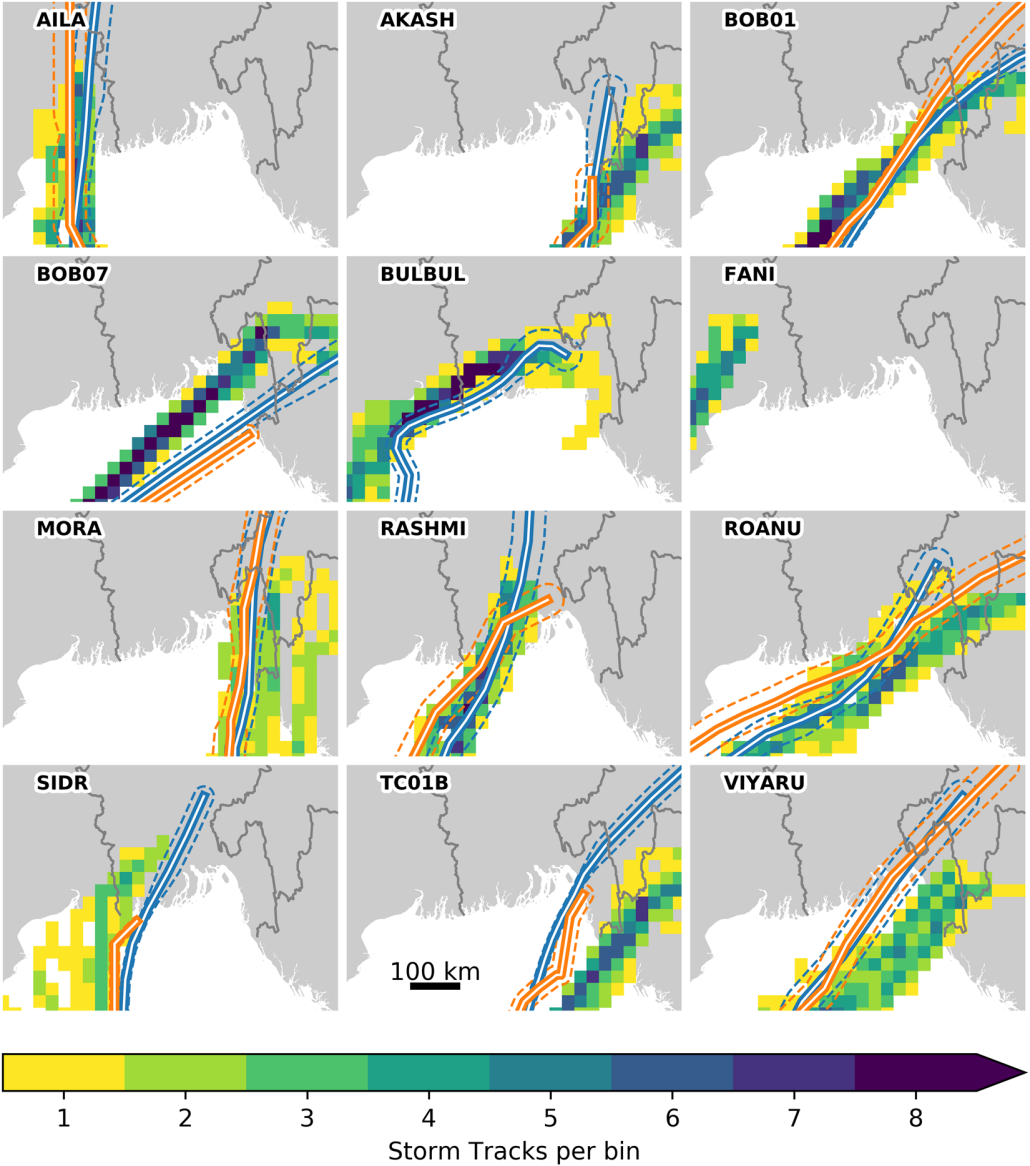
Storm track comparisons for IBTrACS US (blue lines) and ND (orange) with RAL2 ensemble track bin densities. Note that IBTrACS data for the most recent cyclone Fani and Bulbul are incomplete at the time of writing. Dashed lines represent variable IBTrACS storm track uncertainty, based on cyclone intensity.

Comparing storm tracks (Fig. 7) shows that for 8 of 12 cyclones, the RAL2 storm tracks have at least one ensemble member that makes landfall with the bounds of an IBTrACS track. Notable exceptions to this are: BOB07, which shows high consistency in storm track amongst the RAL2 ensemble, but makes landfall to the north of the IBTrACS estimates; TC01B and Viyaru, which show greater spread amongst the RAL2 ensemble members, but consistently make landfall to the south of the IBTrACS estimate. Note that no IBTrACS track data are available for cyclone Fani at the time of writing.

### Differences between 1.5 km and 4.4 km model output

**Fig. 8:**
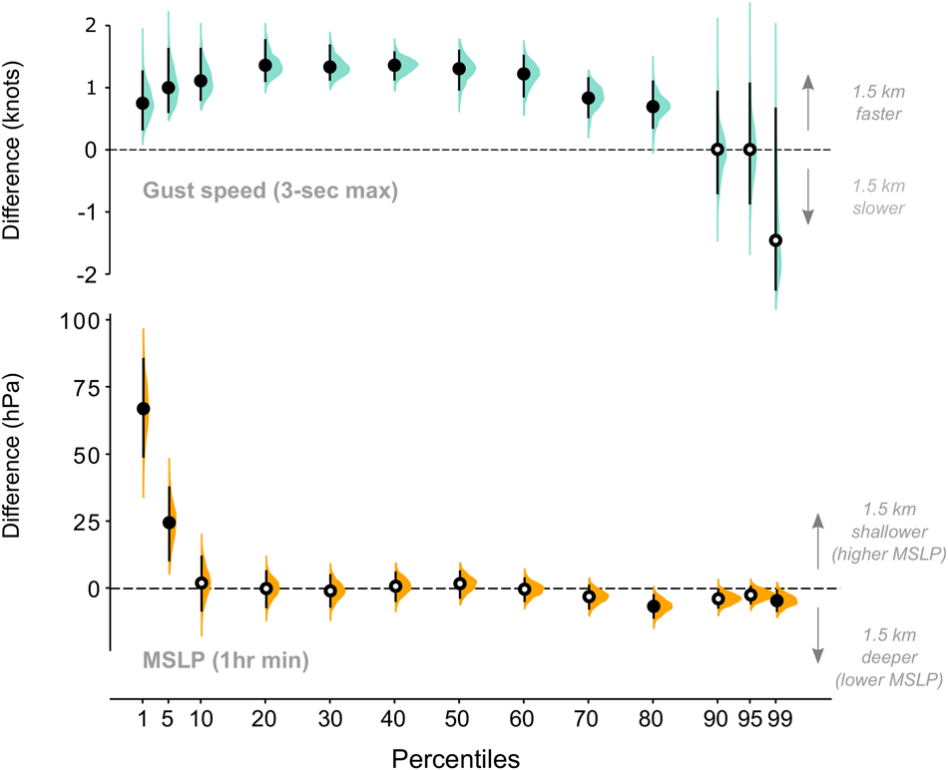
Percentile differences between 1.5 km and 4.4 km tropical cyclone data for (**a**) maximum gust speed and (**b**) minimum mean sea-level pressure (MSLP) footprints. Differences between resolutions are assessed on a quantile basis, in a hierarchical manner to account for dependence between storm ensemble members sampled from multiple storms. Quantile median estimates are shown by black circles, with 95% highest density intervals (HDI) shown by black bars. Where the 95% HDI overlaps 0, the median circles are filled white. The bootstrapped difference distribution (n = 1000) at each quantile is shaded turquoise (gust speeds) and orange (MSLP).

We don’t explicitly validate the 1.5 km data but summarise differences between the distributions of maximum gust speed and minimum MSLP on a quantile basis, in relation to the 4.4 km data (Fig. 8). In order to facilitate a fair comparison, we compare identical spatial domains roughly equivalent to the 1.5 km model domain (see Fig. 2), but with a reduced northern extent to exclude as much mountainous terrain as possible, whilst encompassing the full geographic extent of Bangladesh.

Differences in maximum gust speed footprints, for the 1^st^ to 80^th^ percentiles, of the 1.5 km data are order 1 kn faster than the 4.4 km data. In all cases these differences are sufficiently robust that the 90% HDI of the differences amongst storms does not overlap zero ([0.3, 1.7] kn; [0.14, 0.86] m/s). For the very highest gust speeds (90^th^, 95^th^ and 99^th^ percentiles of the 1.5 km data) the differences with the 4.4 km data shows much greater variability. The 90% HDI does overlap zero, with extremes of the quantile differences ranging from −2.4 kn to 1 kn ([−1.22, 0.50] m/s). Compared to lower percentiles, there are comparatively less data in the extreme upper percentiles, so the large range in this case is expected. Given the relatively robust speed increase seen in the 1.5 km data, compared to the 4.4 km data, for lower percentiles, we suspect that the minimal difference seen in the upper extreme percentiles results from under sampling rather than a systematic difference. Although we might expect the speed increase in the 1.5 km data to be consistent across all percentiles given better sampling, we cannot draw this conclusion based on these 12 storms alone.

For minimum MSLP footprints, the 1^st^ and 5^th^ percentiles of the 1.5 km data are [50, 87] hPa and [10, 37] hPa shallower respectively (90% HDI), but note that the equivalent under sampling observed for high percentiles of gust speeds is likely to be prevalent in the low percentiles of MSLP. All other percentiles do not show any robust differences – the 90% HDI ranges [−11, 12] hPa. We do not feel these results show robust evidence for a systematic difference in MSLP between the 1.5 km and 4.4 km data.

The percentile differences suggest that the environmental MSLP (i.e. high percentiles) on the edge of the cyclone are similar in both the 1.5 km and 4.4 km simulations. Given the relationship between central pressure deficit (i.e. the difference between the tropical cyclone central pressure and the environmental pressure outside the tropical cyclone), peak wind speed and tropical cyclone size^36^, this comparisons suggests that 1.5 km storms may also be smaller in size than the 4.4 km storms. This result is commonly cited in analyses of general circulation models^37–39^ and reanalysis data^40,41^.

In general, the substantial increase in computing effort required to generate 1.5 km gust speed simulations, over and above the 4.4 km simulations, is probably not merited for most catastrophe risk applications given the nature of the parametrisation. Comparing other parameters, such as vorticity or maximum wind speed, may yield different conclusions, and this would be an interesting direction for further research.

### Other notable results

There is a semi-diurnal sea level pressure oscillation which occurs in the days preceding the minimum in MSLP. This oscillation is particularly noticeable in the ERA5 dataset for storms Aila, Bulbul, Rashmi, Roanu, Sidr and Viyaru, and to a lesser extent in RAL2 cyclones Akash, Mora, Rashmi, Roanu and TC01B (see Appendix A). The IBTrACS data does not capture this oscillation, probably due to the limited time sampling. This may be a manifestation of the diurnal radiation cycle as noted by Tang & Zhang^42^, Dunion *et al*.^43,44^ and Knaff *et al*.^45^, amongst others. From simulation studies, Tang & Zhang^42^ in particular note that the absence of a diurnal cycle (principally night time cooling) fails to trigger convection outside the cyclone inner core. Night-time cooling and associated destabilization typically enhance the primary storm vortex, eventually promoting the development of outer rain bands and increasing the size of the storm. Where this process is not evident in model simulations, it could diagnose simulations that have not correctly simulated the cyclogenesis stage and are therefore likely to underestimate cyclone intensity. In our case, for most RAL2 simulations, as shown in the Supplementary Information Figures A1–A11, we do not allocate enough simulation time to the tropical cyclone pre-landfall to make this assessment (for computational efficiency reasons) and we have trimmed the spin-up period from the plots. Assessment of future tropical cyclone simulations could benefit from earlier initialisation times to investigate this further.

It is worth emphasising that the RAL2 model wind speed typically compare more favourably with IBTrACS wind speed data than to ERA5 wind speed. Based on the evaluation of these 12 events, tropical cyclone hazards in the ERA5 deterministic output may underestimate wind and gust intensity, and MSLP depth for tropical cyclones. For some specific cases, despite the ERA5 representation of Fani and Bulbul being less intense compared to the IBTrACS estimates, our RAL2 ensemble has sufficient model freedom (over a 24 hour spin-up period) to develop the ERA5 initial conditions into peak gust and minimum MSLP intensities that have much greater agreement with the IBTrACS data than the ERA5 data. This adds credibility to the spread of the RAL2 model ensembles: where there is substantial RAL2 ensemble spread (e.g. Viyaru or Mora) we suggest this reflects greater atmospheric variability associated with these events, such that the RAL2 ensemble might producing a wider range of counterfactual storm outcomes than would otherwise be seen in the driving reanalysis. Comparing these event ensembles with the ERA5 ensemble spread would be an interesting avenue of future work.

### Future work

Further work comparing the spread of RAL2 ensembles with the ERA5 uncertainty information would contextualise the range of variability that is introduced by the RAL2 model ensemble configuration. Additionally, work is needed to identify landfall times based on the RAL2 tracks. Future downscaling simulations would benefit from outputting variables required for tracking at hourly intervals, to facilitate hourly storm tracking.

## Usage Notes

### RAL2 time methods

Time methods are defined by the sampling period of the data and the sampling type applied to this period. The sampling period (or sampling interval) is one of: hourly (T1H), 3-hourly (T3H) or 24-hourly (T24H). The sampling type is one of max (maximum), min (minimum), mean or point. Point sampling is an instantaneous sample taken from the model time-step (which is typically much less than the sample period). Together then, T1Hmax is interpreted as hourly maximum data; T3Hmean is interpreted a 3-hourly mean data, and T1Hpoint are model instantaneous time-step output taken every hour.

In addition to timeseries data, we produce time-aggregated data for each ensemble member. Referred to as event ‘footprints’, variables are aggregated by minima or maxima over the entire time period. These are commonly used within the catastrophe modelling industry.

### RAL2 File naming

Model time-series files are named according to the following convention:

$${\rm{VAR}}{\rm{.TIMEMETHOD}}{\rm{.UMRA2T}}{\rm{.TIMEPERIOD}}{\rm{.NAME}}{\rm{.RES}}{\rm{.nc}}$$

where: VAR is a short variable identifier of the variable contained within the netCDF file; TIMEMETHOD is the time method, specifying if the var is a mean, min, max or point and the period of time over which the mean, min, max or point measure is found (as described above); UMRA2T is an identifier for the Met Office regional model type; TIMEPERIOD is the time period that the data spans, in the form START_END formatted as YYYYMMDD; NAME is the common name of the storm for the given time period; RES is the resolution of the dataset, either 4p4km = 4.4 km or 1p5km = 1.5 km grid size.

Files relating to ensemble footprints have a simpler file naming structure:

$${\rm{fpens}}{\rm{.VAR}}{\rm{.TIMEMETHOD}}{\rm{.NAME}}{\rm{.RES}}{\rm{.nc}}$$

## Supplementary information

Supplementary Information

## Data Availability

The Met Office Unified Model is available for use under licence. Several research organisations and national meteorological services use the UM in collaboration with the Met Office to undertake basic atmospheric process research, produce forecasts, develop the UM code, and build and evaluate Earth system models. For further information on how to apply for a licence, see https://www.metoffice.gov.uk/research/approach/collaboration/unified-model/partnership Python and R code used to process the RAL2 data is available from Zenodo^46^.

## References

[1] C3S. ERA5: Fifth generation of ECMWF atmospheric reanalyses of the global climate. (2017).

[2] Hersbach, H. *et al*. Operational global reanalysis: progress, future directions and synergies with NWP including updates on the ERA5 production status. *ERA Rep. Ser. No. 27*10.21957/tkic6g3wm (2018).

[3] Bonanno R, Lacavalla M, Sperati S (2019). A new high‐resolution Meteorological Reanalysis Italian Dataset: MERIDA. Q. J. R. Meteorol. Soc..

[4] Taddei, S., Capecchi, V., Pasi, F., Vannucchi, V. & Bendoni, M. Downscaling ERA-5 reanalysis data for coastal short-term and long-term risk assessment in the North Western Mediterranean sea. **21**, 18262 (2019).

[5] Wang X, Tolksdorf V, Otto M, Scherer D (2021). WRF‐based dynamical downscaling of ERA5 reanalysis data for High Mountain Asia: Towards a new version of the High Asia Refined analysis. Int. J. Climatol..

[6] Skamarock, W. C. *et al*. A Description of the Advanced Research WRF Version 4. *NCAR Tech. Note* **145**10.5065/D6MK6B4K (2019).

[7] Kaur, M. *et al*. Dynamical downscaling of a multimodel ensemble prediction system: Application to tropical cyclones. *Atmos. Sci. Lett*. **21** (2020).

[8] Srinivas CV, Bhaskar Rao DV, Yesubabu V, Baskaran R, Venkatraman B (2013). Tropical cyclone predictions over the Bay of Bengal using the high-resolution Advanced Research Weather Research and Forecasting (ARW) model. Q. J. R. Meteorol. Soc..

[9] Singh KS, Bhaskaran PK (2020). Prediction of landfalling Bay of Bengal cyclones during 2013 using the high resolution Weather Research and Forecasting model. Meteorol. Appl..

[10] Mahala BK, Mohanty PK, Das M, Routray A (2019). Performance assessment of WRF model in simulating the very severe cyclonic storm “TITLI” in the Bay of Bengal: A case study. Dyn. Atmos. Ocean..

[11] Knapp KR, Kruk MC, Levinson DH, Diamond HJ, Neumann CJ (2010). The International Best Track Archive for Climate Stewardship (IBTrACS). Bull. Am. Meteorol. Soc..

[12] Knapp, K. R., Diamond, H. J., Kossin, J. P., Kruk, M. C. & Schreck, C. J. I. *International Best Track Archive for Climate Stewardship (IBTrACS) Project, Version 4*. 10.25921/82ty-9e16 (NOAA National Centers for Environmental Information, 2018).

[13] Steptoe, H. & Economou, T. Improving our understanding of wind extremes from Bangladesh tropical cyclones: insights from a high-resolution convection-permitting numerical model. *Nat. Hazards Earth Syst. Sci*. submitted 10.5194/nhess-2020-299 (2020).

[14] Brown A (2012). Unified Modeling and Prediction of Weather and Climate: A 25-Year Journey. Bull. Am. Meteorol. Soc..

[15] Bush M (2020). The first Met Office Unified Model–JULES Regional Atmosphere and Land configuration, RAL1. Geosci. Model Dev..

[16] Ullrich PA, Zarzycki CM (2017). TempestExtremes: a framework for scale-insensitive pointwise feature tracking on unstructured grids. Geosci. Model Dev..

[17] Bechtold P, Bidlot J-R (2009). Parametrization of convective gusts. ECMWF.

[18] Kruk MC, Knapp KR, Levinson DH (2010). A Technique for Combining Global Tropical Cyclone Best Track Data. J. Atmos. Ocean. Technol..

[19] IBTrACS. International Best Track Archive for Climate Stewardship (IBTrACS). Technical Documentation. *National Oceanic and Atmospheric Administration, National Climatic Data Center* 1–24 (2019).

[20] WMO. *Tropical Cyclone Operational Plan for the Bay of Bengal and the Arabian Sea*. (World Meteorological Organization, 2018).

[21] Tiedtke M (1989). A Comprehensive Mass Flux Scheme for Cumulus Parameterization in Large-Scale Models. Mon. Weather Rev..

[22] Boutle IA, Eyre JEJ, Lock AP (2014). Seamless Stratocumulus Simulation across the Turbulent Gray Zone. Mon. Weather Rev..

[23] Skamarock WC, Evaluating Mesoscale NWP (2004). Models Using Kinetic Energy Spectra. Mon. Weather Rev..

[24] Leutwyler D, Lüthi D, Ban N, Fuhrer O, Schär C (2017). Evaluation of the convection-resolving climate modeling approach on continental scales. J. Geophys. Res. Atmos..

[25] Owens, R. G. & Hewson, T. D. *ECMWF Forecast User Guide*. *ECMWF*10.21957/m1cs7h (2018).

[26] Lock, A., Edwards, J. & Boutle, I. *Unified Model Documentation Paper 024: The Parametrization of Boundary Layer Processes*. (2019).

[27] Steptoe H (2020). Zenodo.

[28] OASIS LMF. Oasis Loss Modelling Framework v1.7.0. (2020).

[29] Klawa M, Ulbrich U (2003). A model for the estimation of storm losses and the identification of severe winter storms in Germany. Nat. Hazards Earth Syst. Sci..

[30] Huang Z, Rosowsky DV, Sparks PR (2001). Long-term hurricane risk assessment and expected damage to residential structures. Reliab. Eng. Syst. Saf..

[31] Rousselet GA, Pernet CR, Wilcox RR (2017). Beyond differences in means: robust graphical methods to compare two groups in neuroscience. Eur. J. Neurosci..

[32] Rousselet, G. A. & Wilcox, R. R. Reaction times and other skewed distributions:problems with the mean and the median. *PsyArXiv*10.31234/osf.io/3y54r (2019).

[33] Wilcox RR, Erceg-Hurn DM (2012). Comparing two dependent groups via quantiles. J. Appl. Stat..

[34] Wilcox RR, Erceg-Hurn DM, Clark F, Carlson M (2014). Comparing two independent groups via the lower and upper quantiles. J. Stat. Comput. Simul..

[35] Harrell F, Davis CE (1982). A new distribution-free quantile estimator. Biometrika.

[36] Chavas DR, Reed KA, Knaff JA (2017). Physical understanding of the tropical cyclone wind-pressure relationship. Nat. Commun..

[37] Bengtsson L, Botzet M, Esch M (1995). Hurricane-type vortices in a general circulation model. Tellus A Dyn. Meteorol. Oceanogr..

[38] Reed KA, Chavas DR (2015). Uniformly rotating global radiative-convective equilibrium in the Community Atmosphere Model, version 5. J. Adv. Model. Earth Syst..

[39] Shaevitz DA (2014). Characteristics of tropical cyclones in high‐resolution models in the present climate. J. Adv. Model. Earth Syst..

[40] Malakar P, Kesarkar AP, Bhate JN, Singh V, Deshamukhya A (2020). Comparison of Reanalysis Data Sets to Comprehend the Evolution of Tropical Cyclones Over North Indian Ocean. Earth Sp. Sci..

[41] Schenkel BA, Hart RE (2012). An Examination of Tropical Cyclone Position, Intensity, and Intensity Life Cycle within Atmospheric Reanalysis Datasets. J. Clim..

[42] Tang X, Zhang F (2016). Impacts of the Diurnal Radiation Cycle on the Formation, Intensity, and Structure of Hurricane Edouard (2014). J. Atmos. Sci..

[43] Dunion JP, Thorncroft CD, Velden CS (2014). The Tropical Cyclone Diurnal Cycle of Mature Hurricanes. Mon. Weather Rev..

[44] Dunion JP, Thorncroft CD, Nolan DS (2019). Tropical Cyclone Diurnal Cycle Signals in a Hurricane Nature Run. Mon. Weather Rev..

[45] Knaff JA, Slocum CJ, Musgrave KD (2019). Quantification and Exploration of Diurnal Oscillations in Tropical Cyclones. Mon. Weather Rev..

[46] Steptoe H (2020). Zenodo.

