# Supplementary material for: Tropical cyclone simulations over Bangladesh at convection permitting 4.4 km & 1.5 km resolution
Source: Sci Data. 2021 Feb 16;8:62. doi: 10.1038/s41597-021-00847-5 (PMC7886856; doi:10.1038/s41597-021-00847-5)
Supplement: Supplementary file 1 — Supplementary Information [file 41597_2021_847_MOESM1_ESM.pdf]

Tropical cyclone simulations over Bangladesh at convection permitting 4.4 km & 1.5 km resolution: Supplementary Information

Table of Contents

Figure A1 2

Figure A2 3

Figure A3 4

Figure A4 5

Figure A5 6

Figure A6 7

Figure A7 8

Figure A8 9

Figure A9 10

Figure A10 11

Figure A11 12

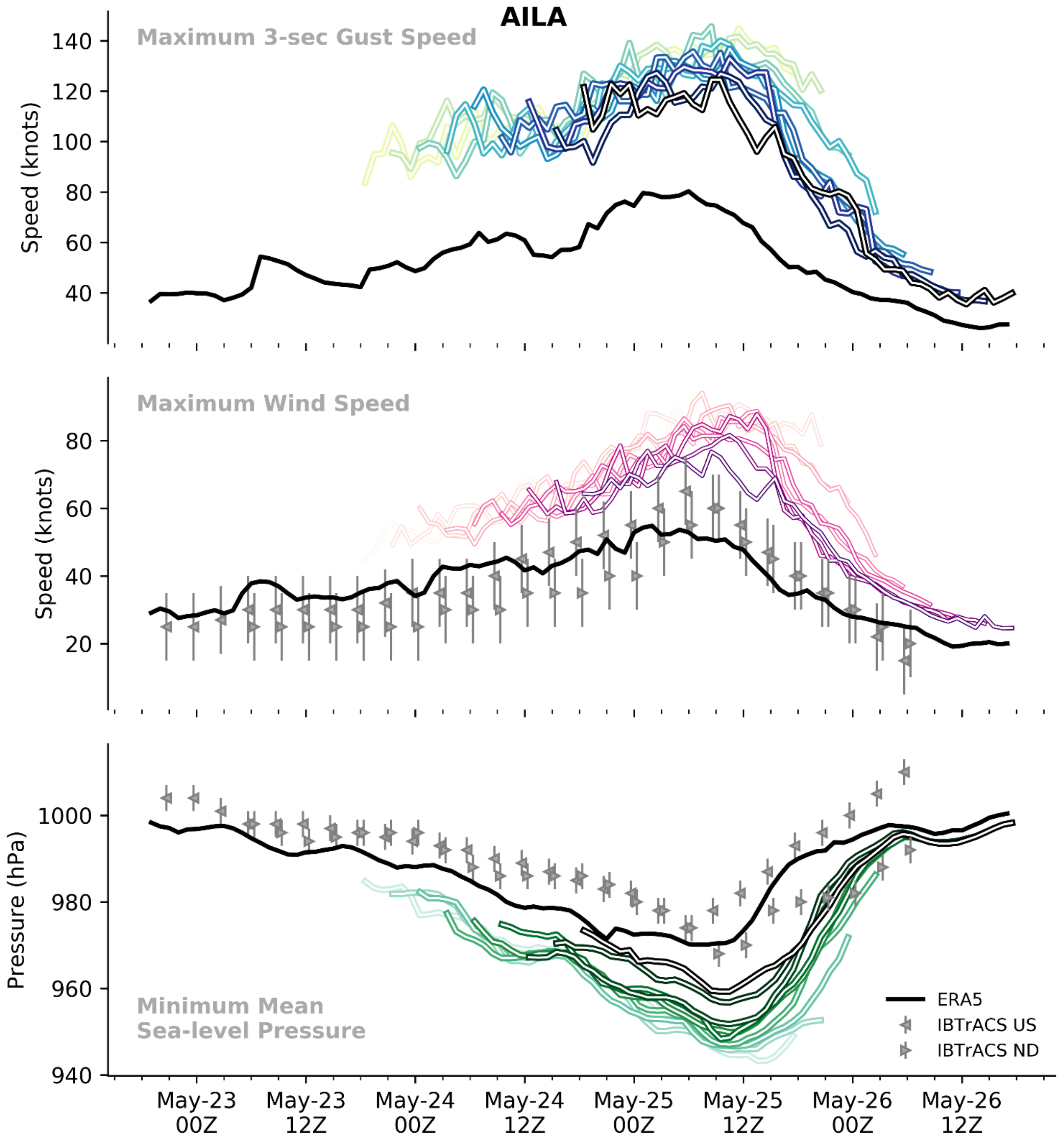

Figure A1 Comparison of maximum wind/gust speed and minimum sea-level pressure for tropical cyclone Aila (May 2009). Details as for Figure 3.

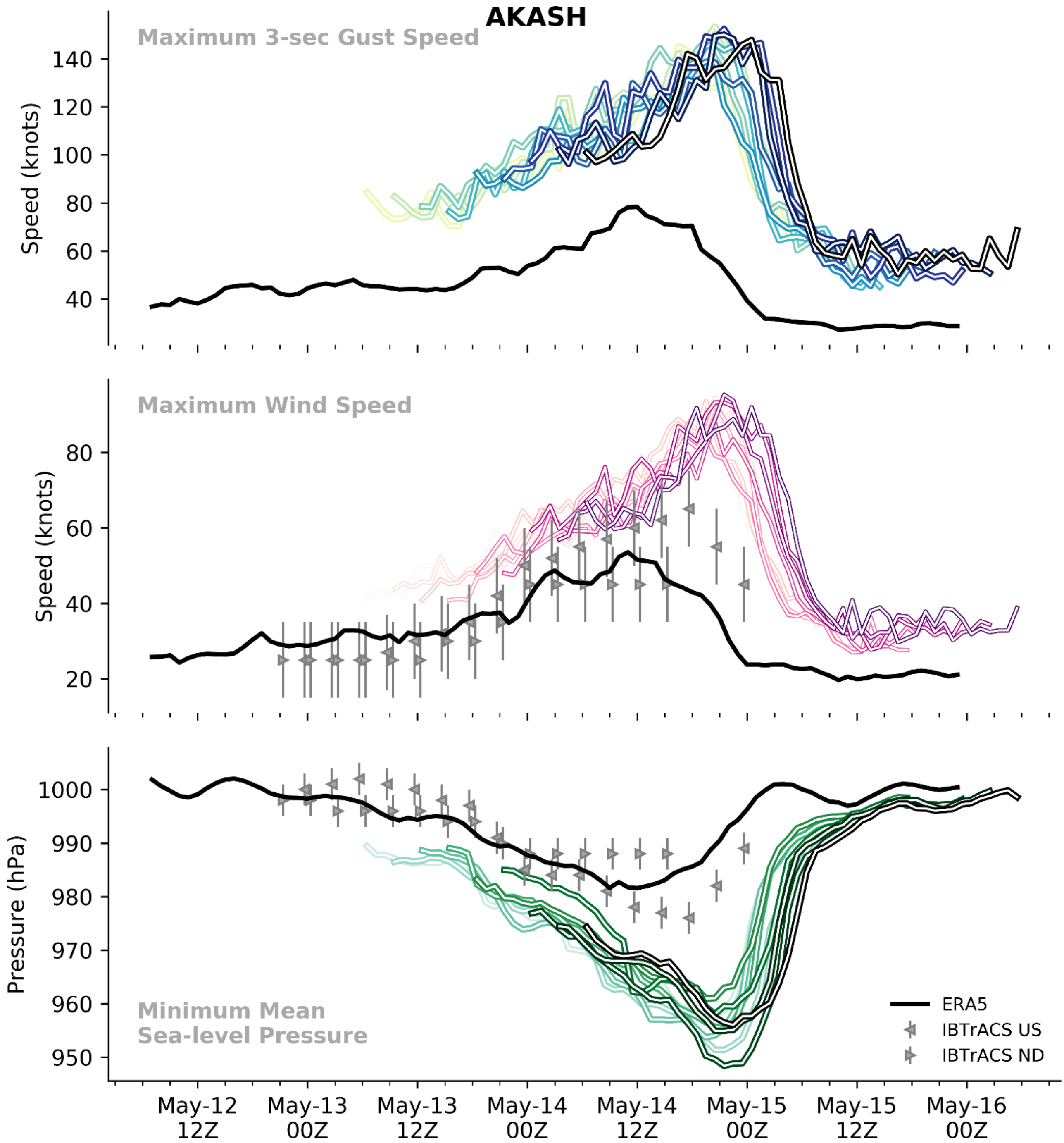

Figure A2 Comparison of maximum wind/gust speed and minimum sea-level pressure for tropical cyclone Akash (May 2007). Details as for Figure 3.

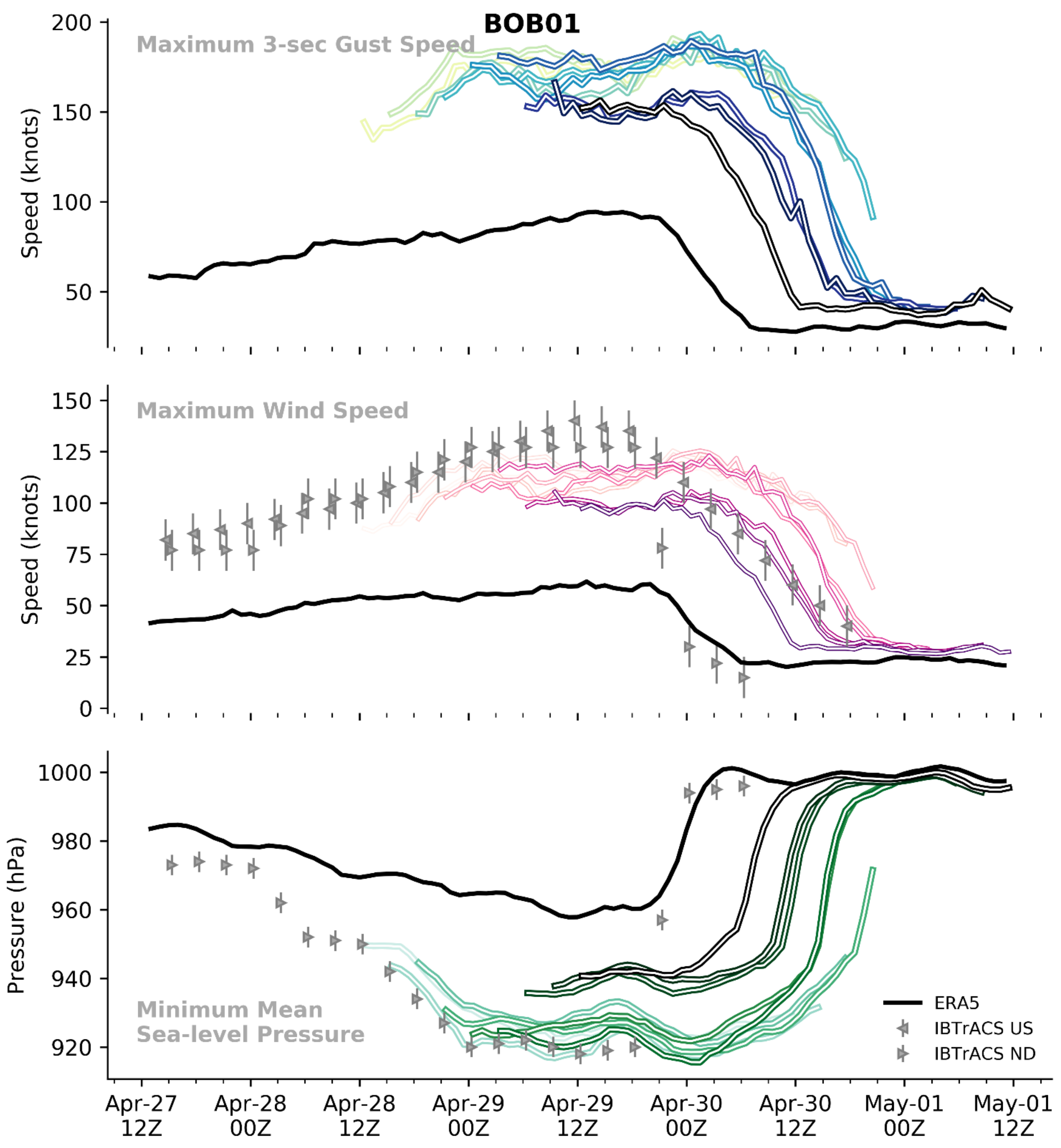

Figure A3 Comparison of maximum wind/gust speed and minimum sea-level pressure for tropical cyclone BOB01 (Apr 1991). Details as for Figure 3.

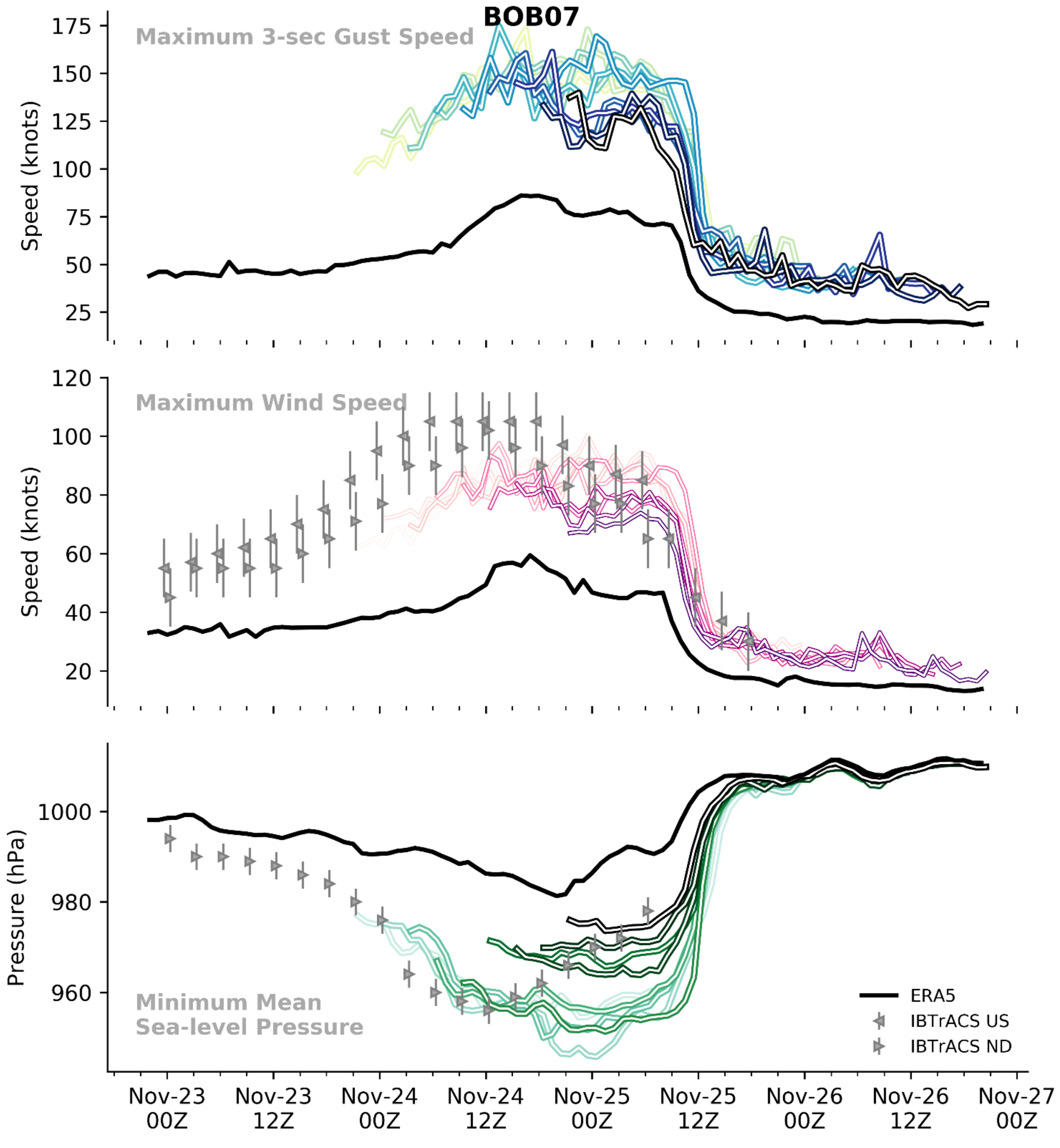

Figure A4 Comparison of maximum wind/gust speed and minimum sea-level pressure for tropical cyclone BOB07 (Nov 1995). Details as for Figure 3.

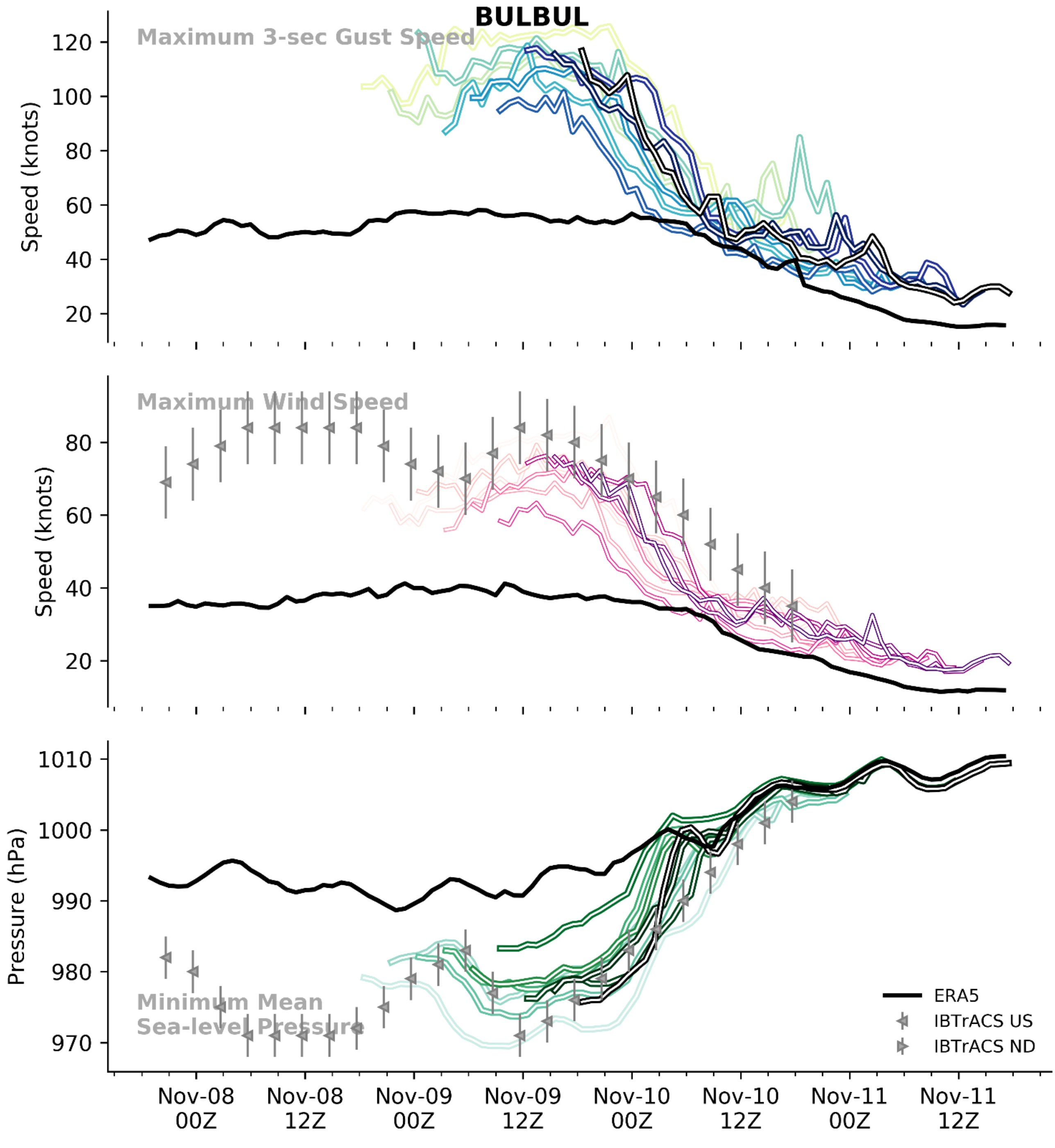

Figure A5 Comparison of maximum wind/gust speed and minimum sea-level pressure for tropical cyclone Bulbul (Nov 2019). Details as for Figure 3.

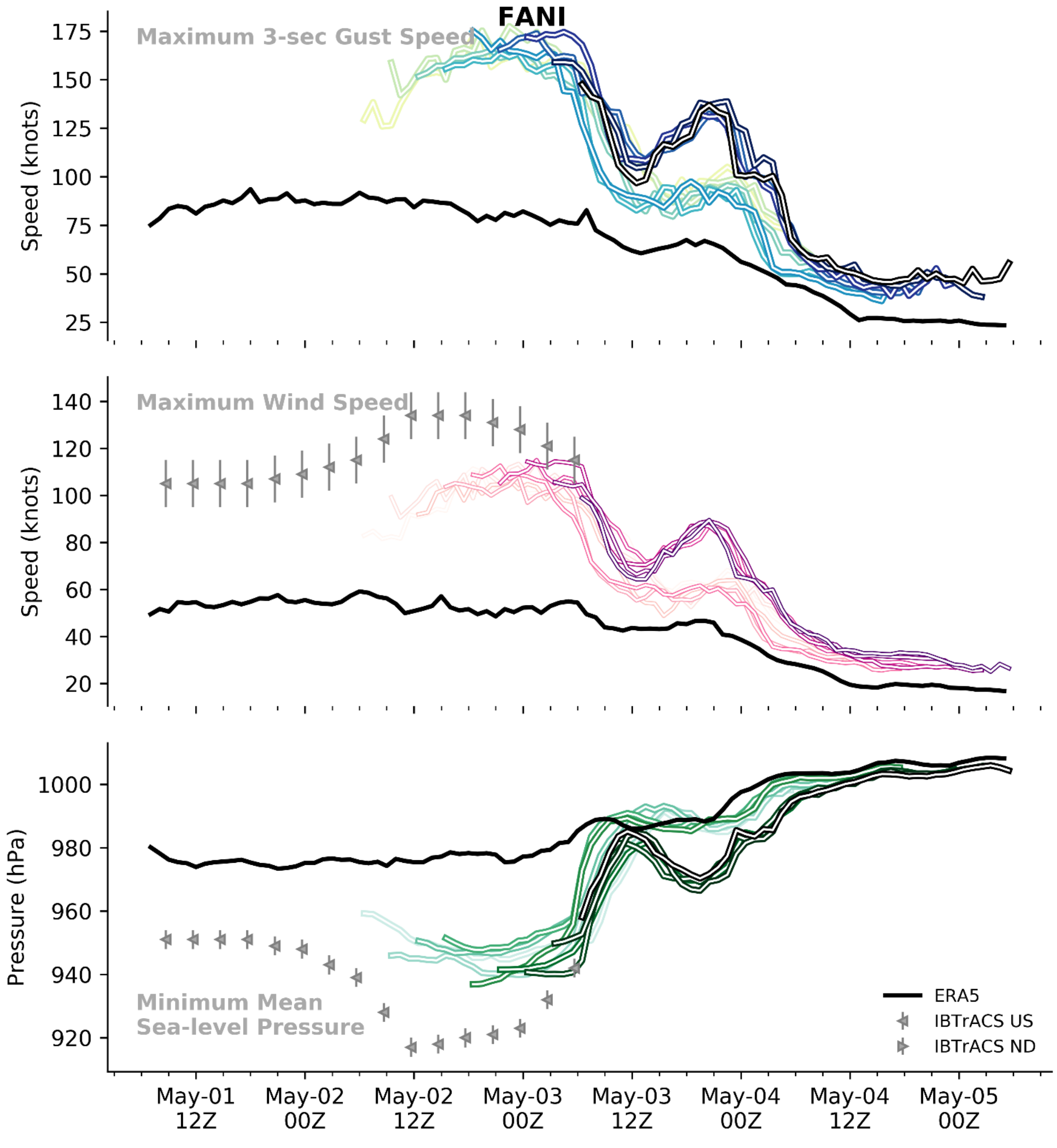

Figure A6 Comparison of maximum wind/gust speed and minimum sea-level pressure for tropical cyclone Fani (May 2019). Details as for Figure 3.

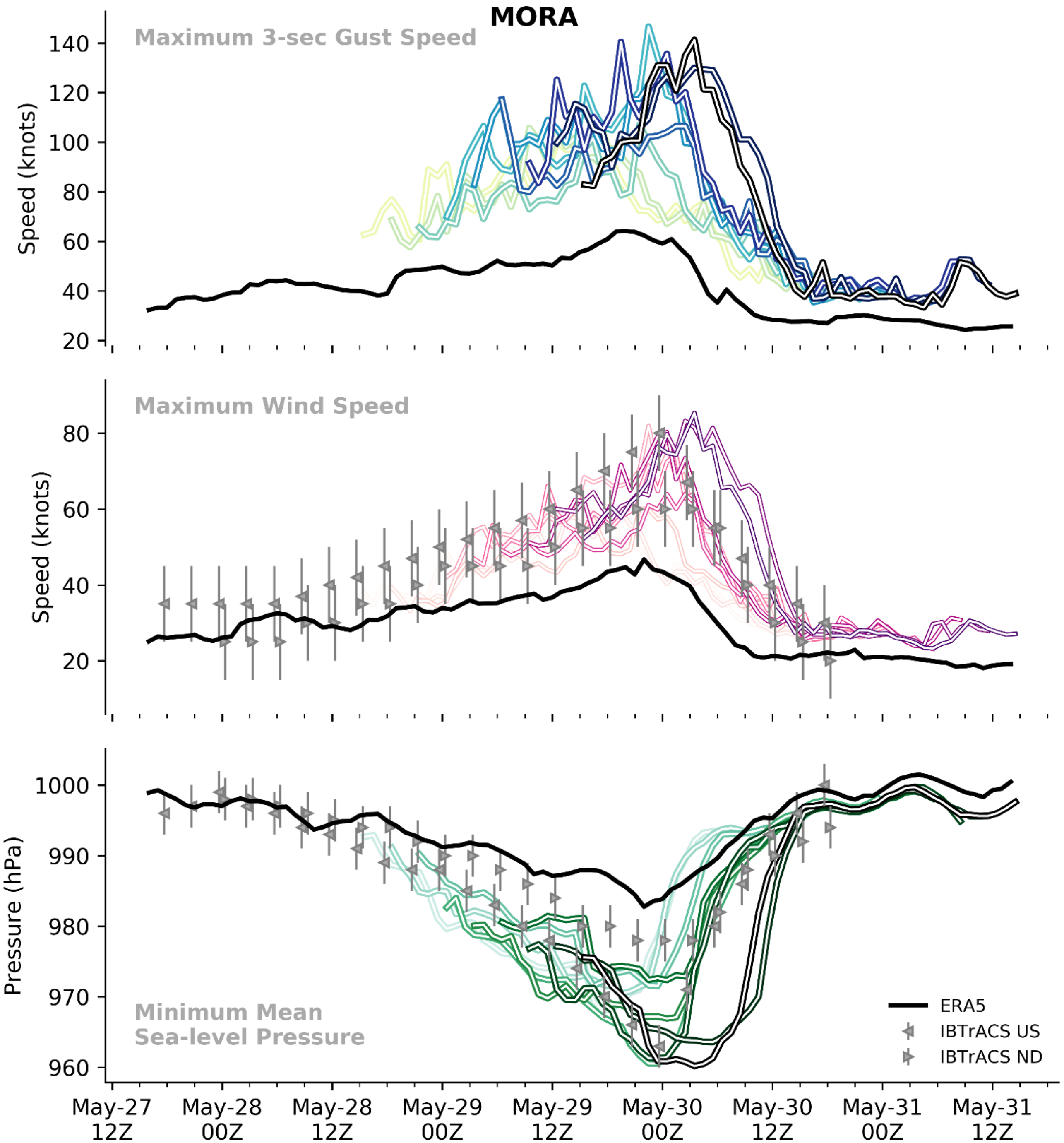

Figure A7 Comparison of maximum wind/gust speed and minimum sea-level pressure for tropical cyclone Mora (May 2017). Details as for Figure 3.

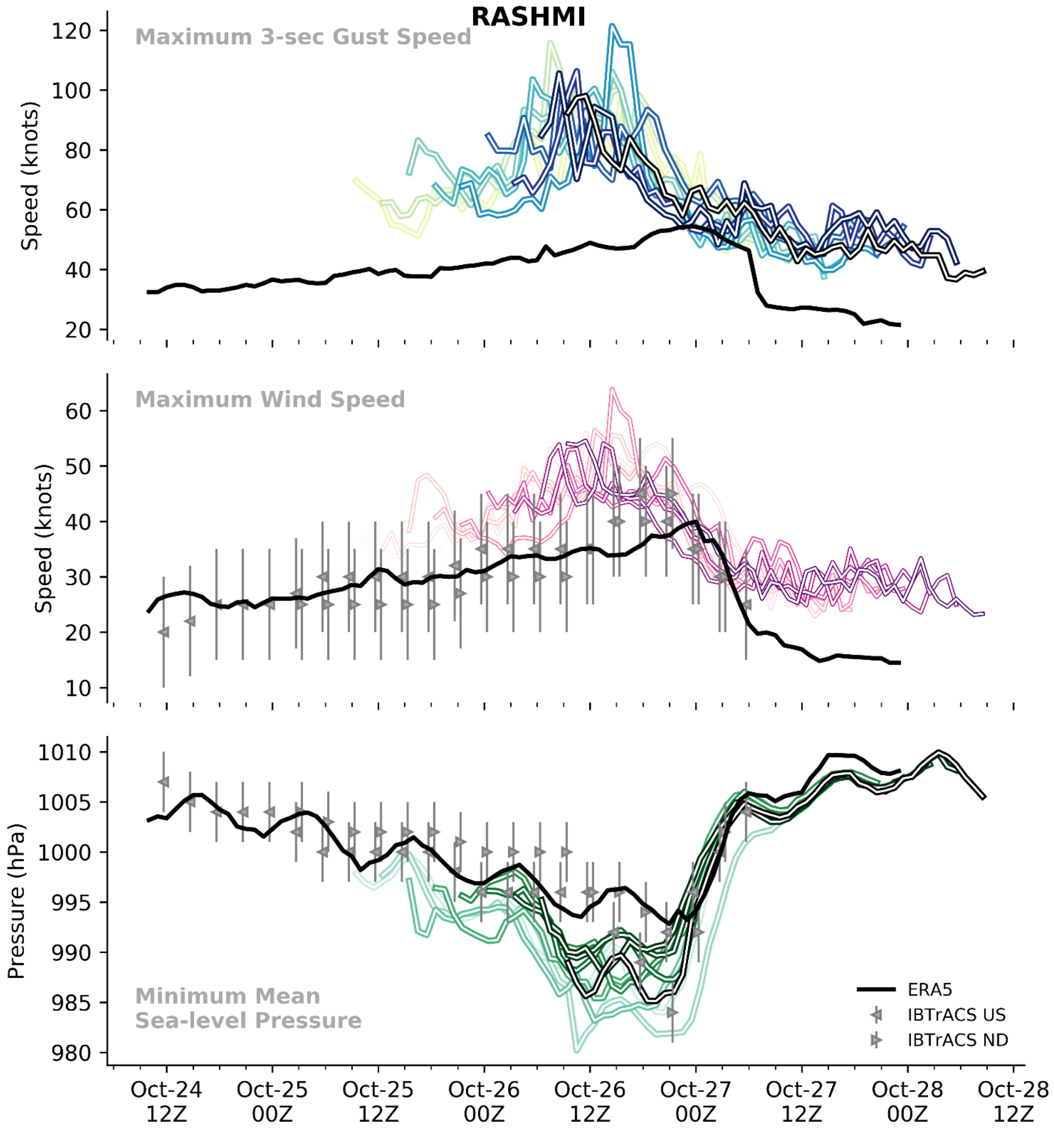

Figure A8 Comparison of maximum wind/gust speed and minimum sea-level pressure for tropical cyclone Rashmi (Oct 2008). Details as for Figure 3.

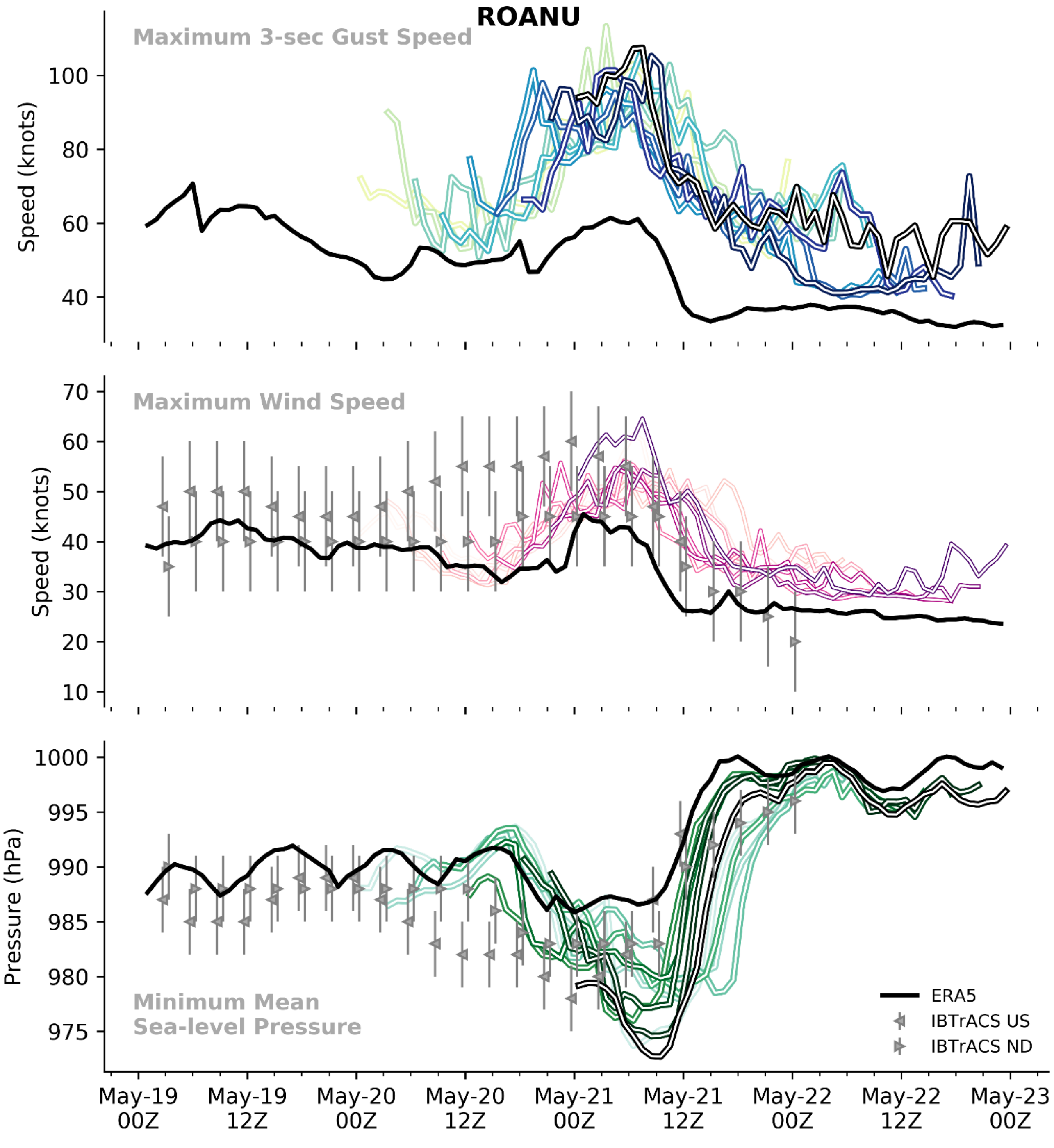

Figure A9 Comparison of maximum wind/gust speed and minimum sea-level pressure for tropical cyclone Roanu (May 2016). Details as for Figure 3.

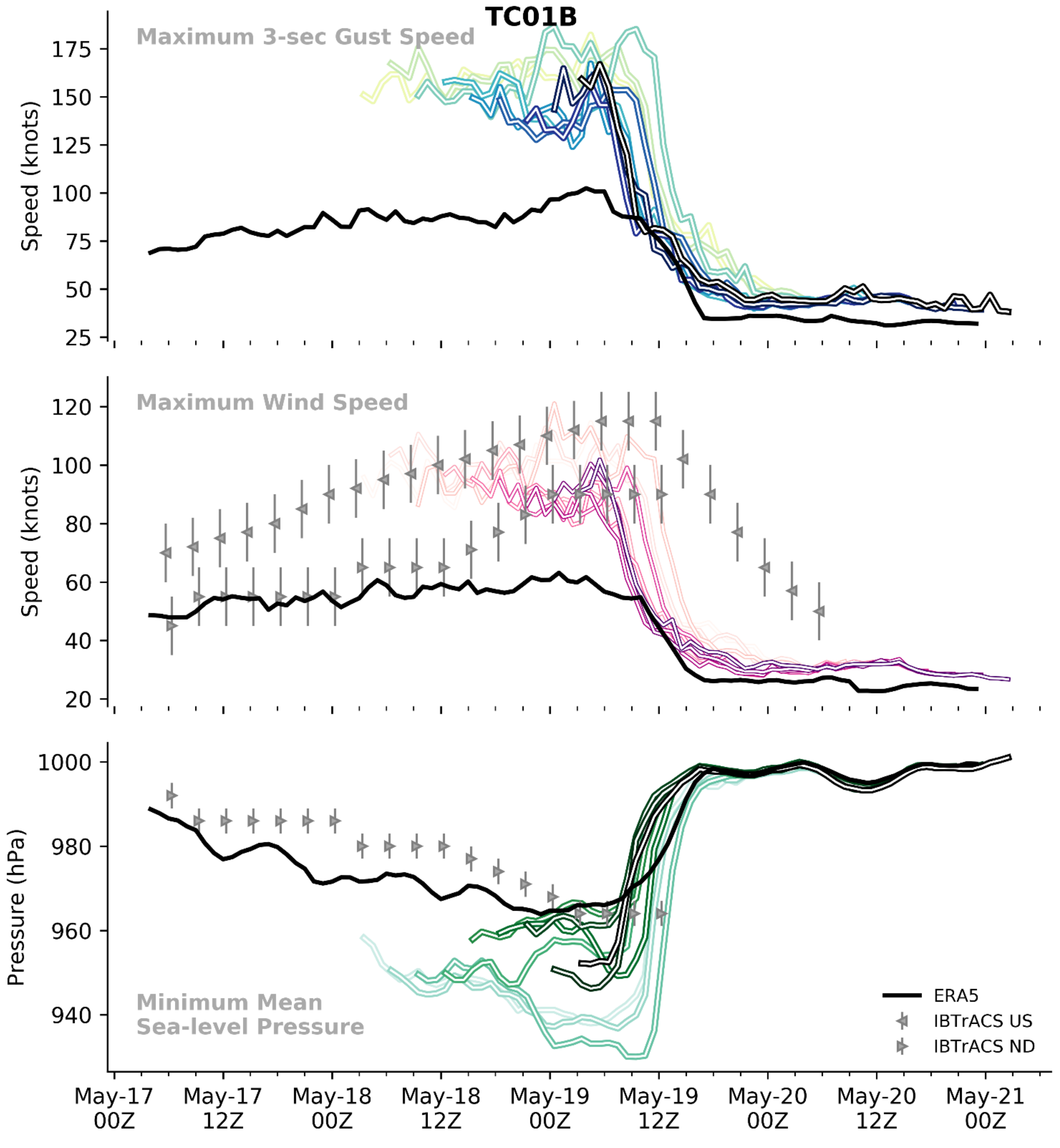

Figure A10 Comparison of maximum wind/gust speed and minimum sea-level pressure for tropical cyclone TC01B (May 1997). Details as for Figure 3.

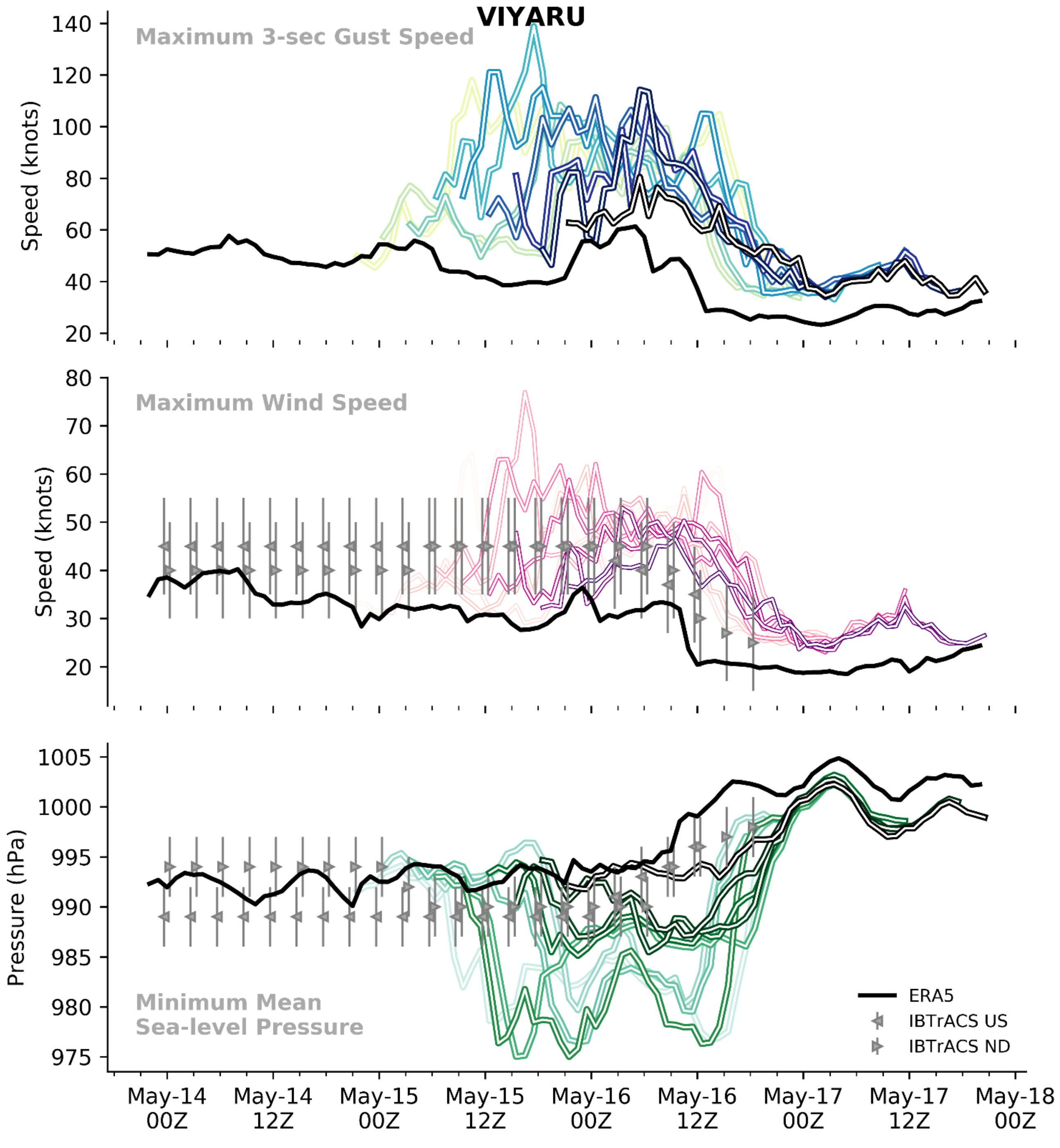

Figure A11 Comparison of maximum wind/gust speed and minimum sea-level pressure for tropical cyclone Viyaru (May 2013). Details as for Figure 3.
